# Lactylated Histone H3K18 as a Potential Biomarker for the Diagnosis and Predicting the Severity of Septic Shock

**DOI:** 10.3389/fimmu.2021.786666

**Published:** 2022-01-06

**Authors:** Xin Chu, Chenyi Di, Panpan Chang, Lina Li, Zhe Feng, Shirou Xiao, Xiaoyu Yan, Xiaodong Xu, Hexin Li, Ruomei Qi, Huan Gong, Yanyang Zhao, Fei Xiao, Zhigang Chang

**Affiliations:** ^1^ Department of Surgical Intensive Care Medicine, Beijing Hospital, National Center of Gerontology, Institute of Geriatric Medicine, Chinese Academy of Medical Sciences, Beijing, China; ^2^ Trauma Center, Department of Orthopaedics and Traumatology, Peking University People’s Hospital, Beijing, China; ^3^ School of Traditional Chinese Medicine, Beijing University of Chinese Medicine, Beijing, China; ^4^ Department of Haematology, Beijing Hospital, National Center of Gerontology, Institute of Geriatric Medicine, Chinese Academy of Medical Sciences, Beijing, China; ^5^ Clinical Biobank, Beijing Hospital, National Center of Gerontology, Institute of Geriatric Medicine, Chinese Academy of Medical Sciences, Beijing, China; ^6^ The Key Laboratory of Geriatrics, Beijing Institute of Geriatrics, Institute of Geriatric Medicine, Chinese Academy of Medical Sciences, Beijing Hospital/National Center of Gerontology of National Health Commission, Beijing, China

**Keywords:** septic shock, critical illness, histone, lactylation, H3K18

## Abstract

**Objective:**

To date, there are no studies regarding the lactylation profile and its role in critically ill patients. Thus, we aimed to examine expression of histone H3 lysine 18 (H3K18) lactylation and its role in patients with septic shock.

**Methods:**

Thirteen healthy volunteers and 35 critically ill patients from the Department of Surgical Intensive Care Medicine, Beijing Hospital were enrolled in our study. Baseline information and clinical outcomes were obtained prospectively. Lactylation levels of all proteins and H3K18 from peripheral blood mononuclear (PBMC) were determined by western blotting and serum levels of inflammatory cytokines by flow cytometry. Arginase-1 (*Arg1*) and Krüppel-like factor-4 (*Klf4*) mRNA expression was evaluated by quantitative real-time PCR (qRT-PCR).

**Results:**

Lactylation was found to be an all-protein post-translational modification and was detected in PBMCs from both healthy volunteers and critically ill patients, with a significantly higher relative density in shock patients (*t*=2.172, *P*=0.045). H3K18la was expressed in all subjects, including healthy volunteers, with the highest level in septic shock patients (compared with non-septic shock patients, critically ill without shock patients and healthy volunteers *P*=0.033, 0.000 and 0.000, respectively). Furthermore, H3K18la protein expression correlated positively with APACHE II scores, SOFA scores on day 1, ICU stay, mechanical ventilation time and serum lactate (*ρ*=0.42, 0.63, 0.39, 0.51 and 0.48, respectively, *ρ*=0.012, 0.000, 0.019, 0.003 and 0.003, respectively). When we matched patients with septic shock and with non-septic shock according to severity, we found higher H3K18la levels in the former group (*t*=-2.208, *P* =0.040). Moreover, H3K18la exhibited a close correlation with procalcitonin levels (*ρ*=0.71, *P*=0.010). Patients with high H3K18la expression showed higher IL-2, IL-5, IL-6, IL-8, IL-10, IL-17, IFN-α levels (*ρ*=0.33, 0.37, 0.62, 0.55, 0.65, 0.49 and 0.374 respectively, *P*=0.024, 0.011, 0.000, 0.000, 0.000 and 0.000 respectively). H3K18la expression also displayed a positive correlation with the level of *Arg1* mRNA (*ρ*=0.561, *P*=0.005).

**Conclusions:**

Lactylation is an all-protein post-translational modification occurring in both healthy subjects and critically ill patients. H3K18la may reflect the severity of critical illness and the presence of infection. H3K18la might mediate inflammatory cytokine expression and *Arg1* overexpression and stimulate the anti-inflammatory function of macrophages in sepsis.

## Introduction

Sepsis, currently defined as a dysregulated immune response to infection, is a systemic inflammatory response induced by infection that can develop into septic shock and even life-threatening organ dysfunction (1, 2). Sepsis represents a major intensive care problem, with incidence rates of up to 535 cases per 100,000 person-years and rising (3). Furthermore, in-hospital mortality due to sepsis remains high at 25-30% (4, 5), and hospital mortality for septic shock ranges from 40 to 60% (6–8). Overall, patients with sepsis and septic shock experience a sharp decline in health-related quality of life during ICU stays and increased rates of death in long-term care (9–11). Despite significant improvements in the diagnosis and management of sepsis, it remains a challenging clinical entity due to its variable aetiology and presentation, and diagnosis is often documented only after clinical deterioration during a hospital stay (12, 13). Differential diagnosis is also difficult in patients with circulatory shock, especially when accompanied by other conditions, such as cardiac injury and hypovolemia and trauma, or in the absence of archetypal signs of infection, e.g., in young infants, the elderly, and the immunocompromised (14–17).

Sepsis initiates a complex immunologic response that dysregulates the homoeostatic balance between pro- and anti-inflammatory processes, and prognosis might be quite different with similar injuries. Recent evidence from the fields of microbiology and immunology, as well as a small number of human sepsis studies, suggests that epigenetic regulation may play a central role in the pathogenesis of this heterogeneous response (18). Indeed, widespread genetic reprogramming leads to disruption of fundamental cellular processes, resulting in endothelial dysfunction, mitochondrial and metabolic derangement, immune failure, and cardiovascular collapse (19), and such events include DNA methylation, histone modifications, and transcriptional regulation by noncoding RNAs (19–21). Histones are subject to a variety of covalent modifications, including methylation, citrullination, acetylation, and phosphorylation, that alter their relationship to each other and to DNA (19). A survival advantage is associated with attenuation of local and systemic proinflammatory cytokines, protection against distant organ injury, enhanced bacterial clearance and phagocytosis, and inhibition of immune cell apoptosis (22, 23). Previous studies found that modifications of histone acetylation and citrullination significantly improve survival, attenuate “cytokine storms” and sepsis-associated coagulopathy, and decrease bone marrow atrophy in a lethal mouse septic model (22–28).

Recently, Zhang *et al.* reported that under biological stress, such as hypoxia, lipopolysaccharides or bacterial infection (such as *Escherichia coli, Acinetobacter baumannii* and *Pseudomonas aeruginosa*), macrophages induce a new post-translational modification (PTM), namely, histone lactylation (29), the discovery of which advances research in this field. The authors found that histones were lactylated in M1 macrophages when exposed to hypoxia, lipopolysaccharide/IFN-γ or bacteria. Increased histone H3 lysine 18 lactylation (H3K18la) induces expression of homeostatic genes involved in healing, including *Arg1.* A previous study showed that p300 [also known as lysine-acetyltransferase (KAT3B)] specifically acetylates histone H3K18 and H3K27 (30). During bacterial and adenovirus infection, H3K18 acetylation is significantly reduced through SIRT2 and CBP/p300 (31). Hence, it is reasonable to hypothesize that the reduction in H3K18 acetylation that occurs in infection may in turn increase H3K18 lactylation (same site of modification) *via* p300, and both may be a promising pair of H3K18 modifications that correlate with sepsis and septic shock.

However, to our knowledge, no clinical study has explored lactylation levels in humans or the relationship between histone lactylation and inflammatory levels during sepsis. Indeed, studies to date have investigated interactions at the cellular level, whereas the expression profile of protein lactylation in humans remains to be investigated; for example, lactylation of histones, which are the major nuclear proteins, has not been examined. In this study, we explored lactylation of all proteins in critically ill patients and further evaluated expression of H3K18la in circulating peripheral blood mononuclear cells (PBMCs) to assess its role in patients with septic shock and in those with non-septic shock. The underlying mechanisms and physiological relevance were further detected through comparisons of inflammatory cytokines and macrophage function biomarkers from the same patients.

## Methods

### Study Design and Participants

This historical cohort study was approved by the institutional ethics board of Beijing Hospital (2018BJYYEC-197-02). Patients admitted to the Department of Surgical Intensive Care Medicine of Beijing Hospital from August 22, 2018, to June 21, 2021, were enrolled. All participants were over 16 years old and signed informed consent; the patients were in the ICU for over 24 hours. Septic shock patients were screened at ICU admission according to the third international consensus definition for sepsis and septic shock (sepsis-3) (2). The definition of septic shock was as follows: vasopressors required to maintain mean artery pressure (MAP)≥ 65 mmHg and serum lactate level ≥ 2 mmol/L despite adequate fluid resuscitation.

Non-septic shock patients included those with haemorrhagic shock, cardiogenic shock, and obstructive shock (pulmonary embolism). The definition of shock includes (32) patients with signs of hypoperfusion and low blood pressure. Tissue hypoperfusion manifests as follows: 1. the skin is cold, clammy and blue, pale or discoloured; 2. altered mental status is present and characterized by obtundation, disorientation and confusion; and 3. urine output is decreased to <0.5 ml/kg/h. Low blood pressure was defined as a systolic blood pressure (SBP) of <90 mmHg, maintenance of a mean artery pressure (MAP) of <65 mmHg, or a decrease of >40 mmHg from baseline.

Critically ill patients without shock were those who did not meet the criteria for shock but were admitted to the ICU for high-risk critical care and intensive treatment, such as major surgery and senior patients with comorbidities.

After enrolment, the following baseline information was collected: age, sex, comorbidities, Sequential Organ Failure Assessment (SOFA) score (ICU admission day one to day three), Acute Physiology and Chronic Health Evaluation II (APACHE II) score within 24 hours, duration of mechanical ventilation, length of ICU stay, length of hospital stay, and 28-day mortality. The following laboratory indicators based from the same collection date were also assessed: serum lactate, white blood cell count (WBC), neutrophil count, neutrophil percentage, lymphocyte count, lymphocyte percentage, monocyte count, monocyte percentage, procalcitonin (PCT) level, and C-reactive protein (CRP) level.

### Blood Samples

Blood samples (5-10 ml) were collected from all participants (ICU patients and healthy volunteers) in Ethylenediaminetetraacetic acid (EDTA)-containing and serum-separating tubes for peripheral blood mononuclear cell (PBMC) isolation and serum separation, respectively. Blood samples were collected within 24 hours after admission.

### PBMC Isolation and Serum Separation

Under sterile conditions, blood samples in EDTA-containing tubes were centrifuged at 3000 rpm for 5 minutes at 20°C. The supernatant was aspirated into cryopreservation tubes and diluted 1:1 with phosphate-buffered saline (PBS) pH-7.2 in a 50-ml tube. The diluted blood samples were layered on top of 15 ml Lymphoprep™ (STEMCELL Technologies Cat# 07851) in a 50-ml tube and centrifuged at 500 x g for 20 minutes at 20°C. Most of the upper layer was aspirated, leaving the white buffy coat at the interphase; the buffy coat was carefully transferred to a new 50-ml tube, which was filled with PBS, mixed and centrifuged at 500 x g for 7 minutes at 20°C. The supernatant was completely removed, and if the precipitate at the bottom of the tubes had red impurities, red blood cell buffer (Solarbio Cat#R1010) was added for 5 minutes; a sufficient amount of PBS was added, and the tube was centrifuged at 500 x g for 7 minutes at 20°C. After removing the supernatant, the PBMCs were harvested and resuspended in 2 ml cryoprotective agent (serum: DMSO = 9:1) and stored at -80°C.

Under sterile conditions, blood samples in serum-separating tubes were centrifuged at 3000 rpm for 10 minutes at 4°C. The supernatant was aspirated into cryopreservation tubes and stored at -80°C.

### Multiple Microsphere Immunofluorescence Assay

Twelve serum cytokines (IL-1β, IL-2, IL-4, IL-5, IL-6, IL-8, IL-10, IL-12p70, IL-17, IFN-α, IFN-γ, TNF-α) were assessed using the multiple microsphere immunofluorescence assay with flow cytometry (cytokines kit, RAISECARE; BD FACS Canto II flow cytometer). The calibration tubes were filled with 25 μl calibration product sample in matrix B, and 25 μl serum sample in buffer was added. The samples were fully mixed with 25 μl of capture microsphere antibodies; then, 25 μl of detection antibodies was added to all tubes, which were shielded from light at room temperature with shaking at 400-500 r/min for incubation. Two hours later, 25 μl SA-PE was added to all tubes, and the tubes were shielded from light at room temperature with shaking at 400-5500 r/min for incubation. Half an hour later, 500 μl of 1× wash buffer was added to the tubes, which were vortexed for several seconds and centrifuged at 500 x g for 5 minutes. After decanting the liquid, 300 μl 1× wash buffer was added to the tubes, which were vortexed for several seconds, and the 12 cytokines indicated above were detected by flow cytometry. At least 1100 microspheres for each sample were collected to ensure the accuracy of the data, which were analysed using LEGENDplex8.0 analysis software.

### RNA Extraction and qRT-PCR

Total RNA was extracted using the guanidine isothiocyanate-phenol-chloroform method (RNAiso Plus, TaKaRa Bio Code No. 9109). The RNA yield was determined by a Thermo Nanodrop 2000C (A260/A280). The quality of the RNA was assessed by agarose gel electrophoresis. Qualified samples were denatured at 65°C for 5 minutes, and reverse transcription of cDNA was conducted with reverse transcription reagents. A DNase step was included to remove residual genomic DNA. The primers used were designed with Primer 5.0 to assemble the upstream and downstream regions of target genes. cDNA and primers were added to a qRT-PCR system (TransStart^®^ Tip Green qPCR SuperMix, AQ141-02; restriction enzymes, Thermo Fisher). The real-time PCR mixture contained 5 µl 2×PCR mix, 0.5 µl primer F (10 µM), 0.5 µL primer R (10 µM), 1 µl template and 3 µl ddH_2_O at a final volume of 10 µl. Reactions were performed in a Roche LightCycler 480 II (Mannheim, Germany) under the following conditions: 95°C for 5 min; 45 cycles of PCR amplification consisting of denaturation at 95°C for 10 sec, annealing at 60°C for 30 sec, and 72°C for 10 sec; and melting curve analysis including 95°C for 5 sec, 65°C for 1 min and 97°C for 1 sec. The samples were cooled at 4°C for 30 sec, and fluorescence was measured. GAPDH was used as an internal control, and expression data were normalized using the delta-delta CT method.

The primers used in qRT-PCR were as follows:

5’-GCTGTGGATGGAAATTCGCC-3’/5’-CTTCTGGCAGTGTGGGTCAT-3’(*Klf4*),
5’-GGGTTGACTGACTGGAGAGC-3’/5’-CGTGGCTGTCCCTTTGAGAA-3’(*Arg1*),
5’-TGACTTCAACAGCGACACCCA-3’/5’-CACCCTGTTGCTGTAGCCAAA3’(*Gapdh*).

### Histone Extraction and Western Blotting

#### Western Blotting With a Pan Anti-Lactyl-Lysine Antibody

Whole-cell lysates were prepared with lysis buffer (1% SDS, 1% protease inhibitors, 3 μM TSA, 50 mM NAM) and sonication. The lysis mixture was centrifuged at 12000 x g for 10 minutes at 4°C to remove cell debris, and the supernatant was transferred to new tubes. Protein concentrations were determined using a Thermo Scientific™ Pierce™ BCA Protein Assay Kit (Cat#23227). Sodium dodecyl sulfate-polyacrylamide gel electrophoresis (SDS-PAGE) was performed. Based on protein concentration results, 15 μg total protein was mixed with 5 μl 4× loading buffer and 2% SDS to a final volume of 20 μl and separated on an SDS-12% polyacrylamide gel at 15 mA/gel for approximately 15 minutes for stacking and at 35 mA/gel for resolution. Staining was performed using Coomassie Blue (R-250) for 2 hours at room temperature, and the gel was then decolorized. For western blotting, anti-lactyl-lysine antibody (PTM-1401RM; Lot: K111421; 1:1000 dilution in Life Technologies™ Antibody Diluent Reagent Solution cat# 003218) was used as the primary antibody and incubated overnight at 4°C. The secondary antibody was goat anti-rabbit IgG (H+L) (Thermo Pierce, Peroxidase Conjugated, 31460) diluted 1:10,000 in TBS-T with 5% milk at room temperature for 2 hours. Bands were detected quantitatively using VILBER Fusion Solo S.

#### Histone Extraction

Total histone proteins were extracted using an EpiQuik Total Histone Extraction Kit (Cat# OP-0006-100): Tubes with PBMCs were centrifuged at 1000 rpm for 5 mins at 4°C and resuspended in Diluted 1X Prelysis Buffer at 10^7^ cells/ml. The tubes were kept on ice for 10 minutes with gentle stirring and centrifuged at 10,000 rpm for 1 min at 4°C. After removing the supernatant, the cells were resuspended in three-fold volumes (approximately 200 µl/10^7^ cells) of lysis buffer and incubated on ice for 30 minutes. After centrifugation at 12,000 rpm for 5 minutes at 4°C, the supernatant (containing acid-soluble proteins) was transferred to new cryopreservation tubes, with 0.3 volumes of balance-DTT buffer added immediately. Protein concentrations were determined using a Thermo Scientific™ Pierce™ BCA Protein Assay Kit (Cat#23227). The isolated histones were stored at −80°C until use.

#### Western Blotting With an Anti-H3k18la Antibody

Histones extracted from PBMCs were assessed for protein concentration using a Thermo Scientific™ Pierce™ BCA Protein Assay Kit (Cat#23227). Based on the protein concentration results, 15 μg total protein was combined with 5 μl 5× loading buffer and 2% SDS to a final volume of 20 μl and separated on a 5% laminated glue + 15% separation gel at 80 V/gel for stacking and 120 V/gel for resolution. Wet transfer to a 0.2-μm PVDF membrane (Immobilon™-P^SQ^ membrane) was performed at 200 mA for 3 hours. The membranes were soaked in blocking buffer (5% skimmed milk) for 2 hours. Primary antibodies were applied overnight at 4°C [anti-lactyl-histone H3 (Lys18) rabbit mAb (PTM-1406RM; Lot: K111421; 1:1000 dilution in Life Technologies™ Antibody Diluent Reagent Solution cat# 003218) and anti-histone H3 antibody Nuclear Marker and ChIP Grade (ab1791) (1:1000 dilution in Life Technologies™ Antibody Diluent Reagent Solution)]. The secondary antibody [goat anti-rabbit IgG H&L (HRP) (ab6721)] diluted 1:3000 in TBS-T with 5% milk was added and incubated at room temperature for 2 hours. The bands detected were quantitatively analysed using VILBER Fusion Solo S.

### Statistical Analysis

Normally distributed data were compared using Student’s *t*-test or one-way analysis of variance (one-way ANOVA), and the results are shown as the mean ± SD; Pearson correlation was applied. Nonnormally distributed data were analysed using the non parametric Mann-Whitney *U* test, and the results are expressed as the median and interquartile range (IQR); Spearman correlation was applied. Categorical variables were compared with the chi-square or Fisher’s exact test, and the results are shown as numbers and percentages. The diagnostic value was determined by receiver operating characteristic (ROC) curve analysis. True positive rate (sensitivity) is plotted against the false positive rate (1-specificity) at different classification thresholds. The area under the ROC curve (AUC) gives an index of the performance of the classifier. Higher values of AUC correspond to a good prediction of the model. *P*<0.05 was considered statistically significant. IBM SPSS 22.0 software was used for all statistical analyses.

## Results

### Baseline Characteristics

The study population included 24 patients with different kinds of shock (including 13 with septic shock, 7 with haemorrhagic shock, 2 with obstructive shock, and 2 with cardiogenic shock), 11 critically ill patients without shock, and 13 healthy volunteers. The mean age and percentage of males were 65.77 years old and 69.2% for the septic shock group and 72.82 years old and 45.5% for the non-septic shock group, 64.27 years old and 45.5% for the critically ill without shock group, and 26.00 years old and 38.5% for the healthy volunteer group, respectively. There were no significant differences in sex or age among the three groups (septic shock, non-septic shock and critically ill without shock) (*P*=0.517; *P*=0.433). There was also no significant difference in comorbidities among the three groups (more details in **Table 1**). Compared to the group of critically ill patients without shock, patients in the shock groups (septic shock and non-septic shock) had higher SOFA and APACHE II scores (*P*=0.001; *P*=0.000) and longer ICU stays and mechanical ventilation times (*P*=0.000; *P*=0.009); however, there were no significant differences in hospital stay between these two groups (*P*=0.295). One patient in the septic shock group and 2 in the non-septic shock group died during the study period.

**Table 1:** Baseline characteristic of ICU patients and healthy volunteers.

Baseline Characteristics	ALL (n = 48)	Septic Shock (n = 13)	Non-septic Shock (n = 11)	Critically ill without Shock (n = 11)	Healthy Volunteers (n = 13)	*P* value in the first three groups	*P* value in four groups
		Mean ± SD/Median (IQR)					
Age (years)	62.00 (28.00-77.75)	65.77 ± 16.65	72.82 ± 12.45	64.27 ± 19.43	26.00 (24.50-28.00)	0.433	0.000
Sex [male (%)]	24 (50.00)	9 (69.23)	5 (45.45)	5 (45.45)	5 (38.46)	0.517	0.518
Comorbidities [n ](%)							
Chronic Pulmonary Disease	3 (6.25)	1 (7.69)	0 (0.00)	2 (18.18)	0 (0.00)	0.497	0.287
Chronic Kidney Disease	3 (6.25)	1 (7.69)	0 (0.00)	2 (18.18)	0 (0.00)	0.497	0.287
Cardiovascular Disease	10 (20.83)	3 (23.08)	4 (36.36)	3 (27.27)	0 (0.00)	0.894	0.101
Hepatopathy	3 (6.25)	2 (15.38)	1 (9.09)	0 (0.00)	0 (0.00)	0.760	0.486
Diabetes	10 (20.83)	4 (30.77)	2 (18.18)	4 (36.36)	0 (0.00)	0.724	0.083
Hypertension	18 (37.50)	7 (53.85)	4 (36.36)	7 (63.64)	0 (0.00)	0.409	0.002
Hyperlipidemia	3 (6.25)	0 (0.00)	1 (9.09)	2 (18.18)	0 (0.00)	0.279	0.122
Malignant Tumor	15 (31.25)	4 (30.77)	5 (45.45)	6 (54.55)	0 (0.00)	0.576	0.011
APACHE II score	21.00 (16.00-24.00)	28.85 ± 11.02	19.82 ± 4.92	15.18 ± 5.98	–	0.001	–
SOFA score on day 1	7.00 (5.00-10.00)	11.08 ± 3.52	7.46 ± 2.07	4.00 (3.00-7.00)	–	0.000	–
Length of ICU stay (days)	7.00 (3.00-19.00)	20.00 (10.00-56.00)	5.00 (3.00-10.00)	2.00 (1.00-4.00)	–	0.000	–
Length of Hospital stay (days)	24.00 (16.00-47.00)	47.15 ± 34.57	31.82 ± 24.14	21.00 (17.00-32.00)	–	0.295	–
Mechanical ventilation percentage	2 (4.17)	0 (0.00)	0 (0.00)	2 (18.18)	–	0.185	–
Mechanical ventilation time	2.00 (1.00-12.00)	5.00 (2.75-73.00)	2.00 (1.25-10.50)	1.00 (1.00-1.50)	–	0.009	–
28-days mortality	3 (6.25)	1 (7.69)	2 (18.18)	0 (0.00)	–	0.497	–

ICU, intensive care unit; IQR, inter quartile range; APACHE Ⅱ score, acute physiology and chronic health evaluation Ⅱ score; SOFA, sequential organ failure assessment; P values were calculated by Mann-Whitney U test, Students’ t-test or one-way analysis of variance (one-way ANOVA), and χ² test or Fisher’s exact test, as appropriate. P values below 0.05 indicates statistical significance.

### Level of All-Protein Lysine Lactylation

**Figure 1:**
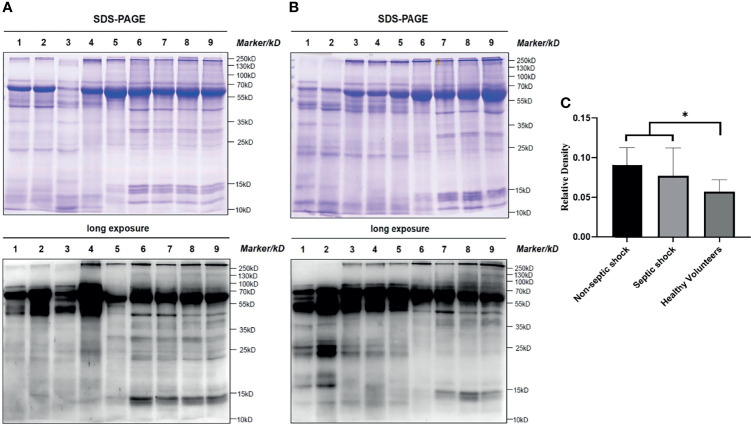
All protein lactyl-lysine lactylation in different groups. Lactylation is an all-protein-posttranslational modification found in both healthy and shock patients. The difference was significant between shock patients and healthy volunteers (Students’ *t*= 2.172, *P*=0.045). **(A)** and **(B)**: Bands 1-2: non-septic shock patients; bands 3-5: septic shock patients; bands 6-9: healthy volunteers; **(C)**: Relative density: (lane greyscale value of the anti-lactyl-lysine antibody)/(lane greyscale of SDS–PAGE × 15 μg). *P < 0.05.

To obtain a primary overall picture of lactylation in the study subjects, a preliminary analysis was performed by collecting clinical information and blood samples from 4 non-septic shock patients, 6 septic shock patients and 8 healthy volunteers, and levels of all-protein lactylation in PBMCs were determined by western blotting. As shown in **Figures 1A, B**, lactylation was an all-protein post-translational modification found in both healthy and shock patients. A difference in expression between the two groups was clearly detectable, as indicated by differences in corresponding band densities. In the all-protein range, shock patients had higher levels of lactylation than healthy volunteers (*t*=2.172, *P*=0.045) (more details are given in **Figure 1C**), whereas differences between septic and non-septic shock patients were not obvious (*Z*=-1.066, *P*=0.286). Internal reference proteins were not available, as they might also be modified. In our study, equal amounts of 15 μg protein were added to each lane such that WB results would be comparable between participants.

### Level of H3K18 Lactylation in ICU Patients and Healthy Volunteers

**Figure 2:**
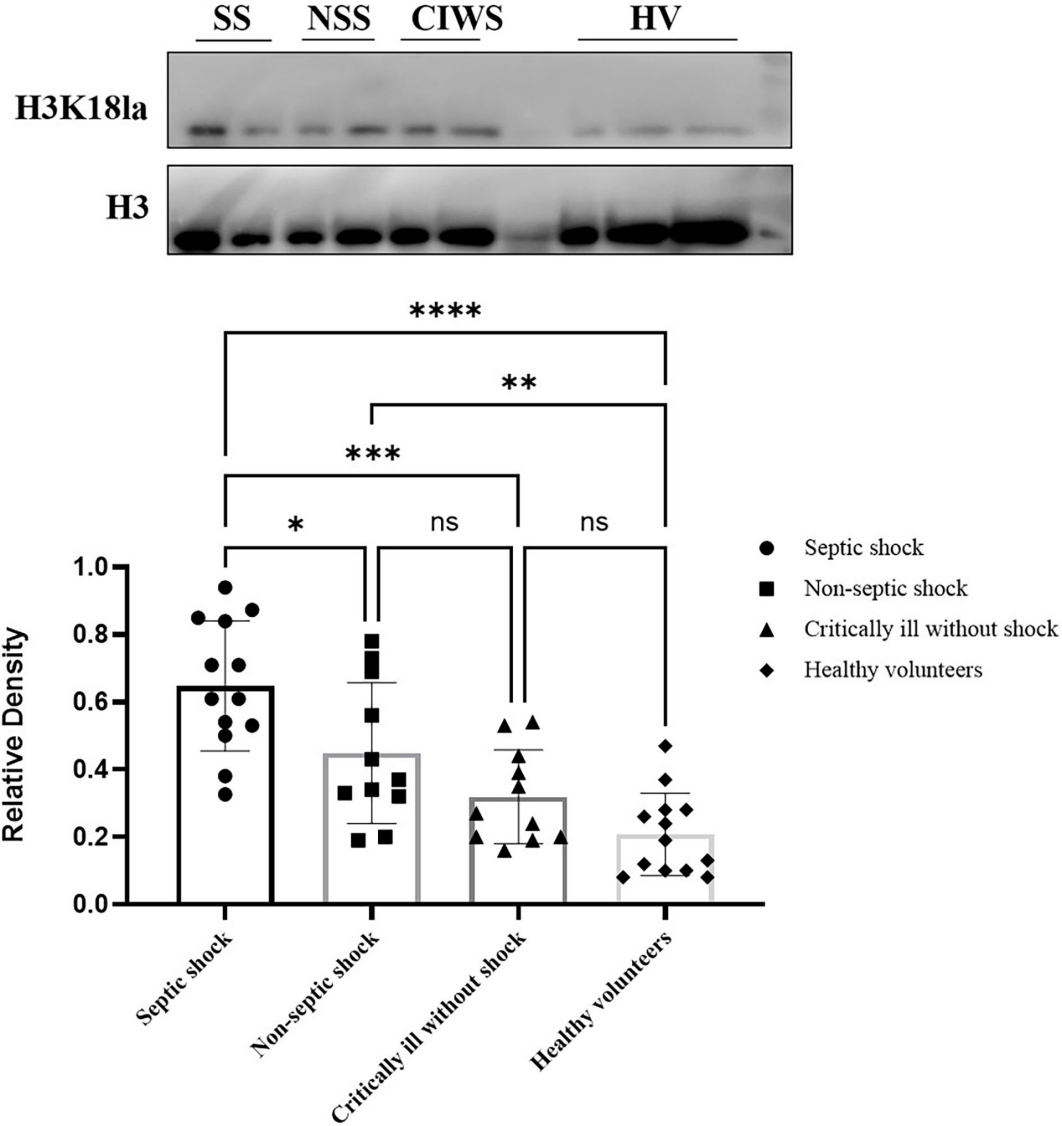
Expression of H3K18 lactylation in ICU patients and healthy volunteers. H3K18la was expressed in all subjects, including the volunteers. The mean level of the H3K18la were significantly different among the four groups of septic shock, non-septic shock, critically ill without shock; and healthy volunteers (Welch ANOVA F=16.158, *P*<0.001). The differences were also significant between the septic shock group and the non-septic group, critically ill without shock group and healthy volunteers (Tukey *post hoc* test *P=*0.033, 0.000 and 0.000, respectively). There is no significant difference between the critically ill without shock group and the healthy volunteers (Tukey *post hoc* test *P=*0.390), or between the non-septic shock group and the critically ill without shock group (Tukey *post hoc* test *P=*0.265). Compared with healthy volunteers, patients in the non-septic shock group have higher H3K18la (Tukey *post hoc* test *P=*0.005). SS, septic shock; NSS, non-septic shock; CIWS, critically ill without shock; HV, healthy volunteers. *P < 0.05, **P < 0.01, ***P < 0.001, ****P < 0.0001, NS, not significant.

H3K18la was expressed in all subjects, including the volunteers (**Figure 2**). The mean level of H3K18la relative density in patients was 0.65, 0.45, and 0.32 in the septic shock, non-septic shock, and critically ill without groups, respectively, and 0.21 in healthy volunteers. Among all ICU patients, H3K18la was highest in those with septic shock (compared with non-septic shock patients, *P*=0.033; compared with critically ill without shock patients, *P*=0.000). Non-septic shock patients also had a higher H3K18la relative density than healthy volunteers (*P*=0.005). However, no significant differences were found between the critically ill patients without shock and non-septic shock (*P*=0.265) or between the critically ill patients without shock and healthy volunteers (*P*=0.390) (more details are given in **Figure 2**).

### H3K18 Lactylation Correlation With Severity and Prognosis

**Figure 3:**
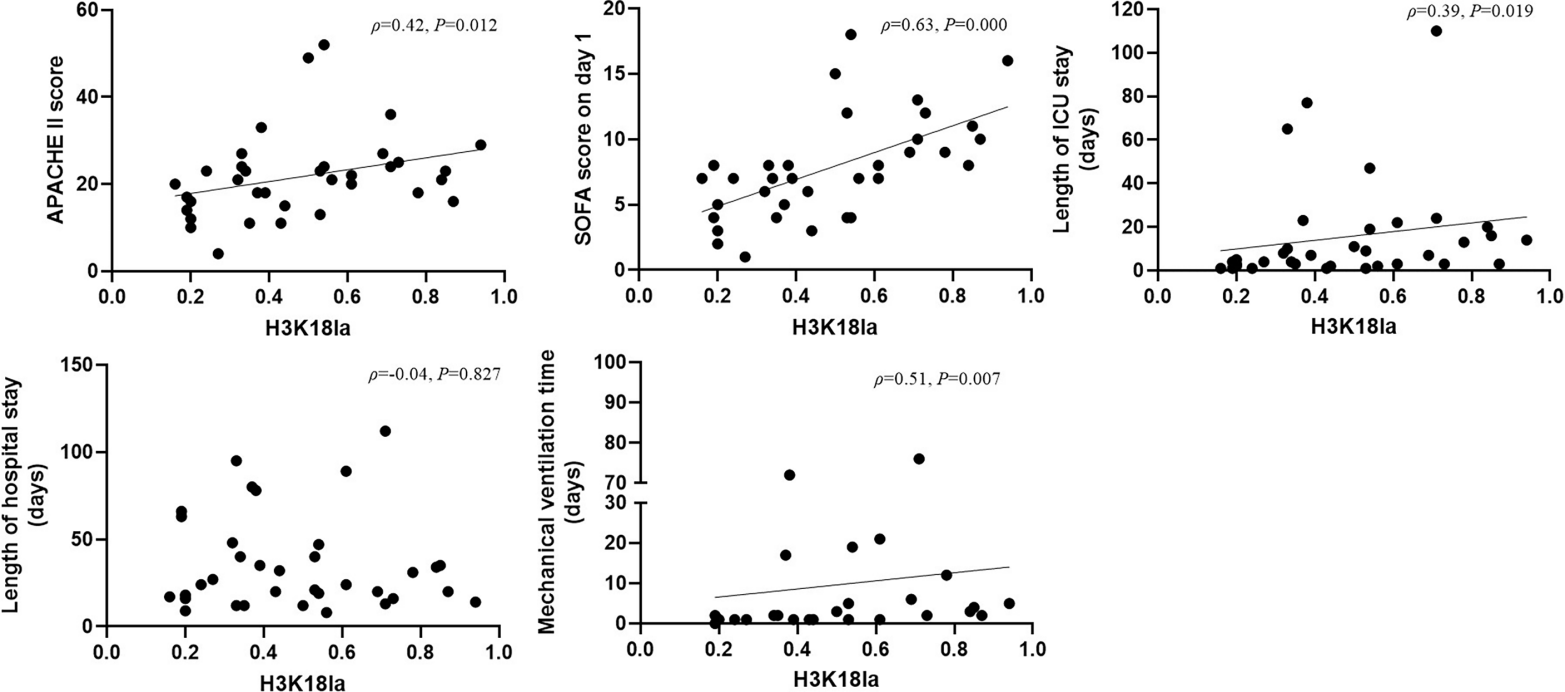
H3K18 lactylation correlation with severity and prognosis. H3K18la has a positive correlation with the APACHE II score, SOFA score on Day 1, length of ICU stay and mechanical ventilation time (Spearman correlation coefficient 0.42, 0.63, 0.39 and 0.51, respectively; *P*=0.012, 0.000, 0.019 and 0.007, respectively). H3K18la, relative density of H3K18 lactylation in western blotting. ICU, intensive care unit; IQR, inter quartile range; APACHE II score, acute physiology and chronic health evaluation II score; SOFA, sequential organ failure assessment. *P* values below 0.05 indicate statistical significance.

There was a positive correlation between H3K18la and the APACHE II score, SOFA score on day 1, ICU stay and mechanical ventilation time (Spearman correlation coefficients 0.42, 0.63, 0.39, 0.51, respectively; *P*=0.012, 0.000, 0.019, and 0.007, respectively) (more details are given in **Figure 3**).

**Figure 4:**
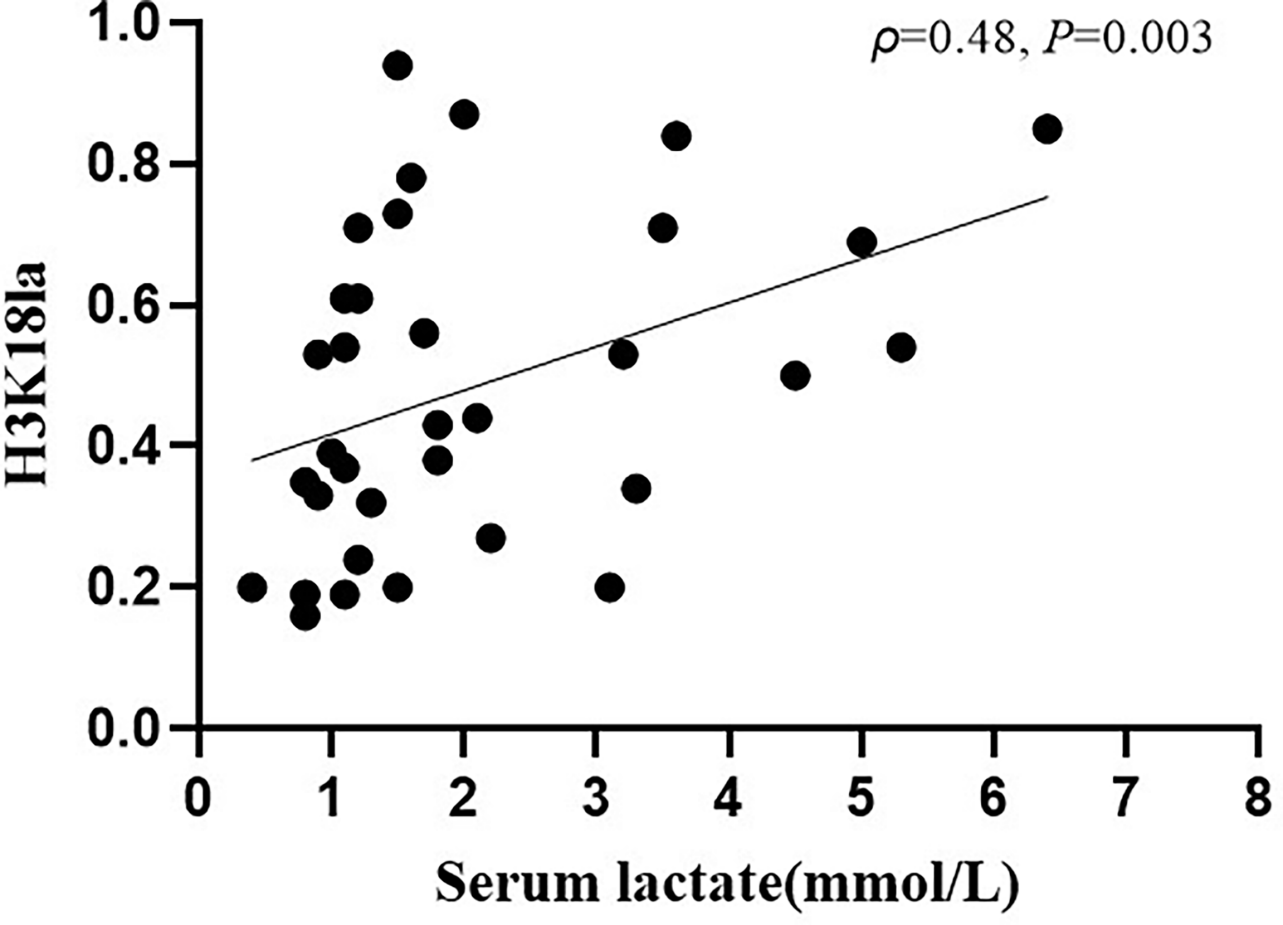
H3K18la lactylation correlation with serum lactate in ICU patients. H3K18la had a positive correlation with serum lactate (Spearman correlation coefficient 0.48; *P*=0.003). H3K18la: The relative density of H3K18 lactylation in western blotting. Serum lactate: Serum lactate level at blood sample date. *P* values below 0.05 indicate statistical significance.

As depicted in **Figure 4**, expression of H3K18la exhibited a positive correlation with the serum level of lactate (Spearman correlation coefficient 0.48; *P*=0.003).

### H3K18 Lactylation Correlation With Infection

As the level of H3K18la correlates with the severity as described above, conclusions should not be drawn simply by comparing relative density in septic shock and non-septic shock patients. As shown in **Supplemental Table 1**, clinical parameters indicating severity and prognosis such as APACHE II, SOFA score on day 1, and ICU stay were significantly different between the septic shock and non-septic shock patients.

To study the role of H3K18la in relation to infection, we matched patients with septic shock and with non-septic shock (haemorrhagic shock, cardiogenic shock and obstructive shock) according to severity and examined prognostic indicators (APACHE II, SOFA, length of ICU stay, and serum lactate). Pairwise comparison of the adjusted median/mean was conducted while taking into account the above parameters. After we removed 3 patients with severe septic shock and 1 non-septic shock patient, there were no significant differences in APACHE II, SOFA score on day 1, ICU stay or serum lactate between the septic shock and non-septic shock patients (*P* =0.141, 0.052, 0.052, and 0.353, respectively; more details are given in **Supplemental Table 2**). We retested H3K18la between septic and non-septic shock patients, and the results still showed a significant difference (*t*=-2.208, *P* =0.040), suggesting that H3K18la is associated with infection. We conducted ROC curve analysis to find out the diagnostic cut-off value of H3K18la in differentiating septic shock patients from non-septic shock patients. A cut-off level of H3K18la relative density over 0.4683 is optimal to make a differential diagnosis, with an 84.6% sensitivity and 63.6% specificity, respectively (more details in **Supplementary Figure 1**).

### H3K18la Lactylation Correlation With Laboratory Parameters of Infection

**Figure 5:**
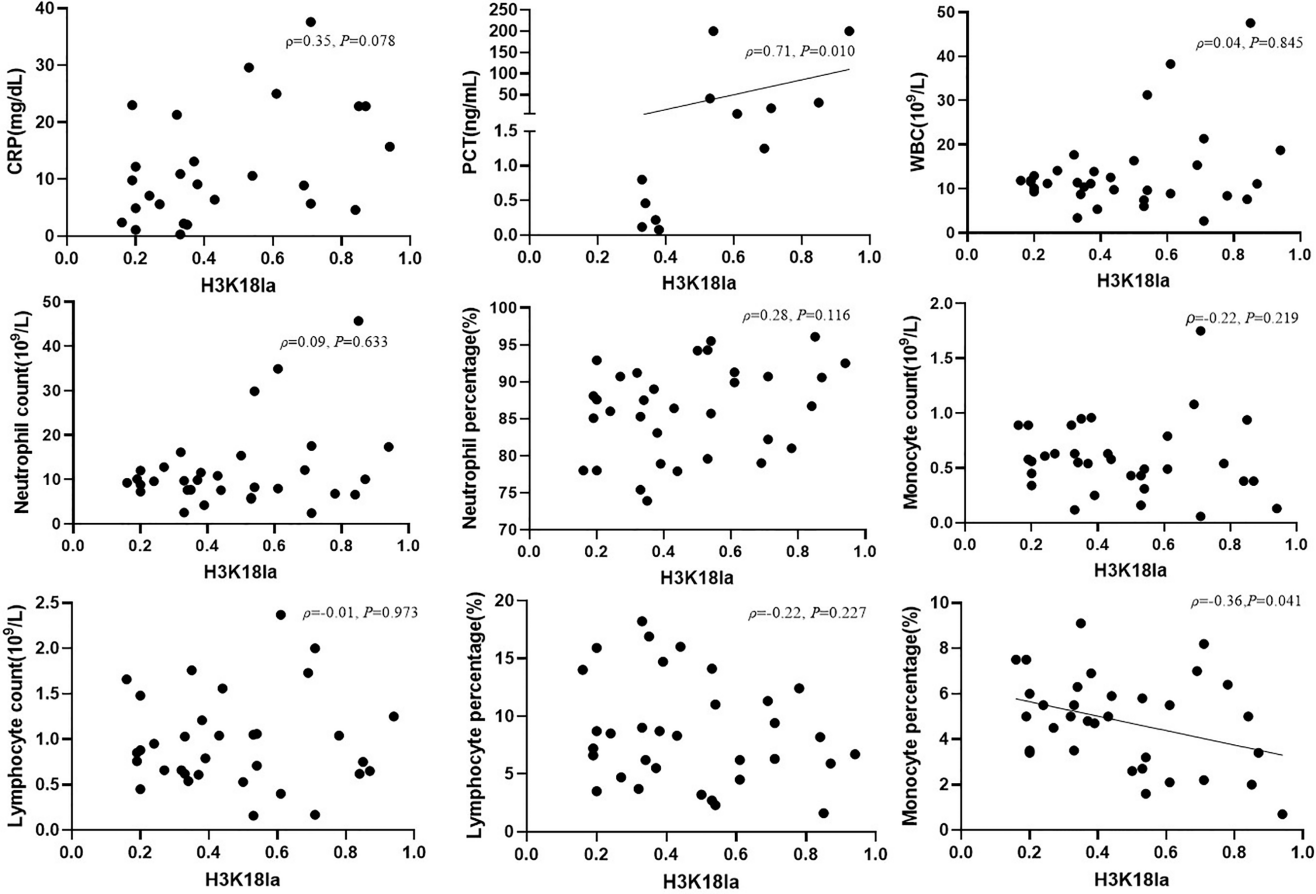
H3K18la lactylation correlation with infectious laboratory parameters. Protein expression of H3K18la has a positive correlation with the serum level of PCT (Spearman correlation coefficient 0.71, *P*=0.010) and has a negative correlation with monocyte percentage (Pearson correlation coefficient -0.36, *P*=0.041). PCT, procalcitonin; CRP, C-reactive protein; WBC, white blood count. *P* values below 0.05 indicate statistical significance.

We further analysed the link between H3K18la expression and inflammatory parameters, including PCT, CRP, WBC, neutrophil count, neutrophil percentage, lymphocyte count, lymphocyte percentage, monocyte count and monocyte percentage, as depicted in **Figure 5**. H3K18la displayed a positive correlation with PCT (Spearman correlation coefficient=0.71, *P*=0.010) but a negative one with the monocyte percentage (Pearson correlation coefficient=-0.36, *P*=0.041).

### H3K18 Lactylation Correlation With Inflammatory Cytokines

**Figure 6:**
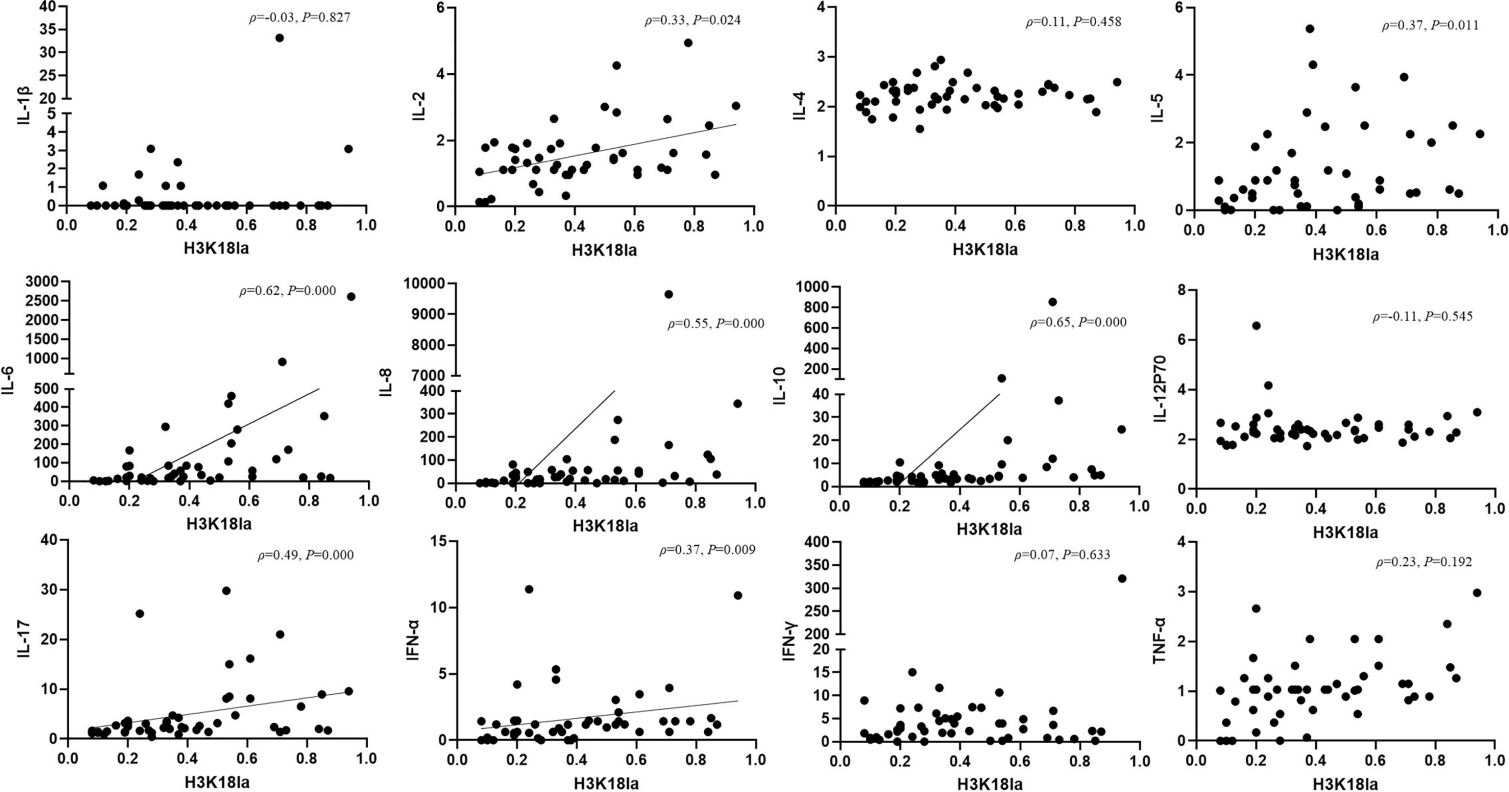
H3K18 lactylation correlation with inflammatory cytokines. Protein level of H3K18la has a positive correlation with the serum levels of IL-2, IL-5, IL-6, IL-8, IL-10, IL-17, IFN-α (Spearman correlation coefficient 0.32, 0.37, 0.62, 0.55,0.65, 0.49 and 0.37 respectively; *P*=0.024, 0.011, 0.000, 0.000, 0.000, 0.000 and 0.009, respectively). H3K18la, relative density of H3K18 lactylation in western blotting. IL, interleukin, IFN, interferon, TNF-tumour necrosis factor. Serum levels of cytokines were assessed using a multiple microsphere immunofluorescence assay with flow cytometry. *P* values below 0.05 indicate statistical significance.

Expression of inflammatory cytokines, including IL-1β, IL-2, IL-4, IL-5, IL-6, IL-8, IL-10, IL-12p70, IL-17, IFN-α, IFN-γ, and TNF-α, was assessed by flow cytometry in all subjects, including healthy volunteers. We further analysed the relationship between H3K18la expression and levels of inflammatory cytokines. As indicated in **Figure 6**, H3K18la exhibited a close positive correlation with IL-6 and IL-10 (Spearman correlation coefficient=0.62 and 0.65, *P*=0.000 and 0.000) and a weak positive correlation with IL-2, IL-5, IL-8, IL-17 and IFN-α (more details in **Figure 6**).

### H3K18 Lactylation Correlation With Markers of Macrophage Function

**Figure 7:**
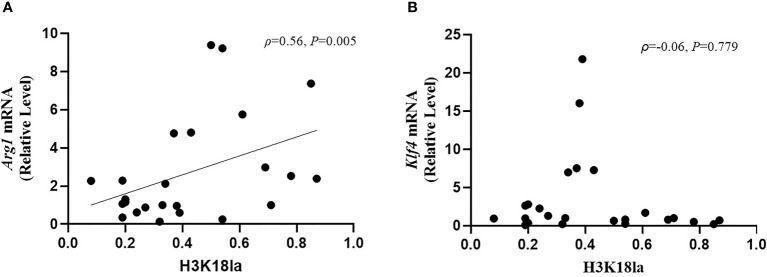
H3K18 lactylation correlation with markers of macrophages function. The protein level of H3K18la had a positive correlation with *Arg1* mRNA expression (Spearman correlation coefficient=0.56, *P*=0.005) but no correlation with *Klf4* mRNA expression (Spearman correlation coefficient=-0.06, *P*=0.779). H3K18la, relative density of H3K18 lactylation in western blotting. qRT-PCR was used to detect *Arg1* **(A)** and *Klf4* **(B)** mRNA expression. GAPDH was used as an internal control.

As illustrated in **Figure 7**, H3K18la had a positive correlation with *Arg1* mRNA expression (Spearman correlation coefficient=0.56, *P*=0.005) but no correlation with that of *Klf4* (Spearman correlation coefficient=-0.06, *P*=0.779).

## Discussion

In our study, we first found that lactylation, a newly identified protein post-translational modification, exists differentially in peripheral blood samples of healthy volunteers and critically ill patients. To our knowledge, this is also the first clinical study exploring the relationship between H3K18la and septic shock. Importantly, our study is the first to indicate that H3K18la correlates significantly with the severity and prognosis of critically ill patients and can differentiate septic shock from non-septic shock.

Lactate, one of the most crucial intermediates of carbohydrate and nonessential amino acid metabolism, has long been mistakenly considered metabolic waste (29). Emerging evidence suggests that lactate is more likely to be a “stress signal”, an autocrine, paracrine and endocrine factor. In acidosis, exogenous lactate infusion has an alkalotic effect (33). As the major gluconeogenesis precursor, lactate serves as fuel (34) and an anti-inflammatory agent in traumatic brain injury (35), acute pancreatitis (36), hepatitis (36), myocardial infarction, cardiac surgery, acute heart failure (37, 38), burns (39) and sepsis (40, 41). In addition, accumulation of lactate helps cancer cells escape immunity and has an inhibitory effect on immune killing (42–46). Following the first report of lactylation in *Nature* in 2019, a series of studies found that macrophages take up extracellular lactate to promote self-protein lactylation under both physiological and pathophysiological conditions (29, 47–50), which further explains the immunological function of lactate at the molecular level.

In our study, we first performed a small-sample-size preliminary experiment and found that lactylation is an all-protein post-translational modification that is present in healthy and ill subjects. We found that lactylation is universally present in humans and on nearly all proteins of various sizes, not only in the range of histones. Additionally, we observed a clearly detectable difference between shock patients and healthy volunteers. Nevertheless, protein degradation could not be avoided. The earliest frozen samples we used were from 2018, and we did not extract histone proteins. We sought to obtain a primary overall picture of lactylation in subjects; the pan anti-lactyl-lysine results could only show an overall expression profile in critically ill patients and healthy volunteers and indicate rough differences in all-protein lactylation.

To date, studies using a pan anti-lactyl-lysine antibody have not found direct evidence demonstrating which histone is lactylated and induces macrophage phenotypic alterations. In 2019, Zhang *et al.* reported that histone lactylation occurs in M1 macrophages exposed to hypoxia, lipopolysaccharide/IFN-γ or bacteria. Increased histone lactylation (H3K18la) induces expression of homeostatic genes involved in healing, including *Arg1*, and is associated with the M2-like phenotype (29). After an extensive literature review, we selected H3K18 as a modification site for detecting lactylation levels in our participants. H3K18la displayed a positive correlation with serum lactate (Spearman correlation coefficient 0.48; *P*=0.003, **Figure 4**). However, as indicated in **Figure 4**, several patients with normal levels of serum lactate (< 2 mmol/l) had a relatively high level of H3K18la, which suggests that H3K18 lactylation might be independent of serum lactate. Our study also shows that H3K18la exhibits a positive correlation with the APACHE II score, SOFA score on day 1, ICU stay and mechanical ventilation time, suggesting that H3K18la may constitute as a prominent independent biomarker that reflects the severity and prognosis of critically ill patients.

Several studies have revealed that lactylation may correlate with the release of inflammatory factors during sepsis (49, 50). p300 (also known as KAT3B), a classic acetyltransferase that specifically acetylates histone H3K18 and H3K27 (30), also catalyses protein lactylation (29), and the study by Eskandarian *et al.* found H3K18 acetylation to be significantly reduced during bacterial and adenovirus infection through SIRT2 and CBP/p300 (31). Overall, it is reasonable to hypothesize that the reduction in H3K18 acetylation in infection may in turn promote H3K18 lactylation (same site of modification) *via* p300. To further explore the role of H3K18la in sepsis, we divided patients with shock into subgroups of septic shock and non-septic shock, and the results showed that the former had the highest level of H3K18la (see details in **Figure 2**). As the clinical outcome of the patients in the septic shock group was worse than that of those in the non-septic shock group (see details in **Supplemental Table 1**), we further conducted pairwise comparisons of adjusted medians/means by taking into account the APACHE II score, SOFA score, ICU stay and lactate level (see details in **Supplemental Table 2**). We re-assessed the H3K18la level between the septic and non-septic shock patients, and the results still showed a significant difference (*t*=-2.208, *P*=0.040), suggesting that H3K18la is involved in the pathophysiologic process of sepsis-induced shock.

Sepsis and septic shock had been associated with multiple biomarkers of inflammation, such as PCT (51, 52), CRP (53), Soluble CD14 subtype (54), and soluble urokinase-type plasminogen activator receptor(suPAR) (55), etc. However, there is no single golden standard diagnostic biomarker to differentiate septic shock patients from non-septic shock patients (2, 56). In our study, we also found an optimal cut-off value of H3K18la relative density (0.4683) to make a diagnosis of septic shock, with an 84.6% sensitivity and 63.6% specificity. Further studies with larger sample size are needed to verify our findings. To further confirm the relationship between H3K18la with infection, we compared expression of H3K18la with clinical biomarkers of infection, including WBC, neutrophil count, neutrophil percentage, lymphocyte count, lymphocyte percentage, monocyte count, monocyte percentage, PCT and CRP levels. The results showed that H3K18la correlates closely with PCT (Spearman correlation coefficient=0.711, *P*=0.010). It is well known that serum levels of PCT are increased in bacterial infection but that levels remain unchanged or only moderately increase in a non-infection condition (57, 58). Combined with our findings, it is reasonable to infer an important relationship between H3K18la and infection.

Regardless, our study did not detect a direct relationship of H3K18la with WBC and CRP. Indeed, the value of leucocytosis in the diagnosis of infection and sepsis remains low, as it is influenced by many non-infectious factors, such as myocardial infarction, catecholamines, and corticosteroids (29), and may also markedly decline in the setting of severe infection (59). Previous studies have reported that PCT has better sensitivity than CRP for differentiating bacterial infections from nonbacterial infections (60, 61), and it has been reported that trauma patients experience various degrees of stress that elicit an inflammatory response, which causes an elevation in PCT concentrations, even in the absence of infection (62). Our study only included one traumatic-haemorrhagic patient, a female aged 81, whose PCT was 0.22 ng/ml, which was within the normal value (<0.5 ng/ml).

Regarding mechanisms of H3K18la in infection, we assessed expression of cytokines and genes that are critical in infection. Zhang *et al.* (29) performed RNA-seq analysis at 0, 4, 8, 16 and 24 h after challenge with LPS and IFN-γ; 1,223 genes specifically marked by increased H3K18la were more likely to be activated or reactivated at later time points (16 or 24 h) during M1 polarization, correlating well with the induction of intracellular lactate and histone Kla levels at these later time points (29). In our study, H3K18la correlated with inflammation-related cytokines, and patients with high H3K18la had higher IL-10 and IL-6 levels (more details are given in **Figure 6**). In the pathophysiology of sepsis, the proinflammatory cytokine IL-6 is known to play a pivotal role and is an overproduced cytokine that causes hypercytokaemia (63). IL-10 signalling activates the Jak-STAT pathway (64) and PI3K-Akt-GSK pathway, a process that suppresses expression of various inflammatory genes (65). IL-10 drives the molecular pathway that enhances immunosuppression during late sepsis (66), which correlates with mortality in patients with infection (67). The results of our analysis of circulating cytokines in patients showed that in addition to proinflammatory cytokines (such as IL-6), concentrations of the potent anti-inflammatory cytokine IL-10 were increased. Combined with the significant relationship with IL-10, H3K18la might not only differentiate patients with sepsis but also indicate prognosis. On the other hand, IL-10 enhances the phenotype of M2 macrophages in synergy with IL-4, which consequently induces expression of anti-inflammatory genes, including *Arg1 (* 68, 69).

*Arg1* and *Klf4* levels increase in macrophages during type 2 immune responses and wound repair (29, 47). Macrophages stimulated by interferon-γ (IFN-γ) and interleukin-4 (IL-4) and IL-10 induce *Arg1* to produce increased amounts of iNOS, inhibiting efficient clearance of bacteria (70). At later time points after infection, H3K18la-mediated anti-inflammatory (such as IL-10 and *Arg1*) overexpression may be related to late death (71). Our results are consistent with current advances involving cellular and molecular findings.

Finally, there are several limitations to this study. First, the clinical findings could not provide direct evidence demonstrating the participation of lactate-induced H3K18la in the regulation of IL-10 in sepsis. Therefore, the effect of sepsis-derived lactylation on the initiation and progression of sepsis remains an open and interesting question. Second, this was a one-centre, small-sample-size, historical cohort study, and several biases were inevitable. Limited by the sample size, the conclusions must be viewed as preliminary and treated with caution, and further studies with larger sample sizes are needed to verify our findings. Lactylation might be a potentially very important mechanism during various biological stresses. As yet, the key enzymes (write, read and erase multiple histone modification), the correlation with acetylation and other modifications, the site-specific gene functions, signalling receptors and pathways remain mostly undefined. In the future, multi-center, large sample size studies are needed to confirm its expression profile in different disease specific patterns, while much more basic researches are needed regarding its mechanisms.

In conclusion, lactylation is an all-protein posttranslational modification that exists in healthy and ill patients. H3K18la may reflect the severity of critical illness as well as the existence of infection. H3K18la correlates positively with inflammatory cytokine production. H3K18la-mediated anti-inflammatory effects, such as IL-10 overexpression, may play an important role in the anti-inflammatory function of macrophages as well as *Arg1* expression in sepsis.

## Data Availability Statement

The datasets presented in this study can be found in online repositories. The names of the repository/repositories and accession number(s) can be found in the article/**Supplementary Material**.

## Ethics Statement

The studies involving human participants were reviewed and approved by the institutional ethics board of Beijing Hospital (2018BJYYEC-197-02). Written informed consent to participate in this study was provided by the participants’ legal guardian/next of kin.

## Author Contributions

XC and ZC contributed to the conception of the study, data interpretation and drafted the manuscript. CD, PC, LL, ZF, SX, XY, XX, HL, RQ, HG, YZ, and FX contributed to data collection and critically revised the manuscript for important intellectual content. All authors contributed to the article and approved the submitted version.

## Funding

This work was supported by the Beijing Hospital New-star Plan of Science and Technology (BJ-2018-134) and Fundamental Research Funds for the Central Universities (3332018173).

## Conflict of Interest

The authors declare that the research was conducted in the absence of any commercial or financial relationships that could be construed as a potential conflict of interest.

## Publisher’s Note

All claims expressed in this article are solely those of the authors and do not necessarily represent those of their affiliated organizations, or those of the publisher, the editors and the reviewers. Any product that may be evaluated in this article, or claim that may be made by its manufacturer, is not guaranteed or endorsed by the publisher.

## Supplementary Material

Supplementary Figure 1 ROC curve of H3K18la for diagnosis septic shock patients from non-septic shock patients. The area under the ROC curve (AUC) was 0.755 (*P* = 0.034). The cut-off value of H3K18la was 0.4683, with sensitivity of 84.6% and specificity of 63.6%. ROC, receiver operating characteristic.
