# Supplementary figures and images for: Lactylated Histone H3K18 as a Potential Biomarker for the Diagnosis and Predicting the Severity of Septic Shock
Source: Front Immunol. 2022 Jan 6;12:786666. doi: 10.3389/fimmu.2021.786666 (PMC8773995; doi:10.3389/fimmu.2021.786666)

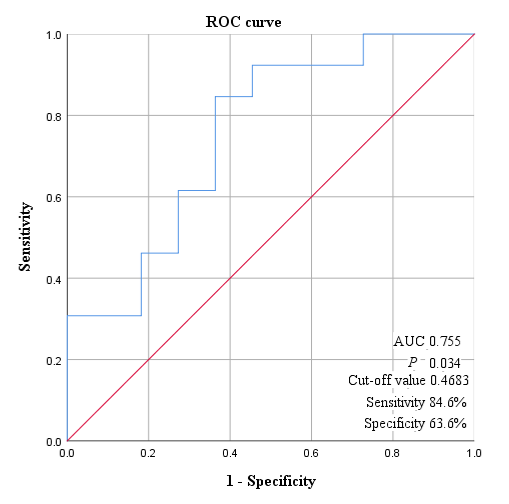

Supplement: Supplementary Figure 1 — ROC curve of H3K18la for diagnosis septic shock patients from non-septic shock patients. The area under the ROC curve (AUC) was 0.755 (P = 0.034). The cut-off value of H3K18la was 0.4683, with sensitivity of 84.6% and specificity of 63.6%. ROC, receiver operating characteristic. [file Image_1.png]
