# Supplementary material for: Lactylated Histone H3K18 as a Potential Biomarker for the Diagnosis and Predicting the Severity of Septic Shock
Source: Front Immunol. 2022 Jan 6;12:786666. doi: 10.3389/fimmu.2021.786666 (PMC8773995; doi:10.3389/fimmu.2021.786666)
Supplement: Supplementary file 2 [file Table_1.docx]

**Supplemental Table 1. Clinical outcome in different shock patients**

| **Clinical outcome** | **Septic Shock**  **(n=13)** | **Non-septic Shock**  **(n=11)** | ***P* value** |
| --- | --- | --- | --- |
|  | Mean ± SD / Median (IQR) | |  |
| APACHE II score | 28.85±11.02 | 19.82±4.92 | 0.020 |
| SOFA score on day 1 | 11.08±3.52 | 7.46±2.07 | 0.007 |
| Length of ICU stay (days) | 20.00(10.00-56.00) | 5.00(3.00-10.00) | 0.000 |
| Length of hospital stay (days) | 47.15±34.57 | 31.82±24.14 | 0.229 |
| Mechanical ventilation time(days) | 5.00(2.75-73.00) | 2.00(1.25-10.50) | 0.009 |
| Serum lactate(mmol/L) | 2.00(1.20-4.05) | 1.50(0.90-1.80) | 0.070 |

ICU, intensive care unit; IQR, inter quartile range; APACHE Ⅱ score, acute physiology and chronic health evaluation Ⅱ score; SOFA, sequential organ failure assessment; *P* values were calculated by Mann-Whitney U test, Students’ t-test or one-way analysis of variance (one-way ANOVA), as appropriate. *P* values below 0.05 indicates statistical significance.

**Supplemental Table 2. Clinical outcome in different shock patients after matched**

| **Clinical outcome** | **Septic Shock**  **(n=10)** | **Non-septic Shock**  **(n=10)** | ***P* value** |
| --- | --- | --- | --- |
|  | Mean ± SD / Median (IQR) | |  |
| APACHE II score | 20.70±4.16 | 23.80±4.83 | 0.141 |
| SOFA score on day 1 | 9.00(8.00-12.25) | 7.50(5.75-9.00) | 0.052 |
| Length of ICU stay (days) | 18.00(7.50-34.25) | 6.00(3.75-10.75) | 0.052 |
| Length of Hospital stay (days) | 44.20±31.30 | 33.00±25.11 | 0.389 |
| Mechanical ventilation time(days) | 5.00(2.25-59.25) | 2.00(2.00-12.00) | 0.397 |
| Serum lactate(mmol/L) | 1.65(1.18-3.30) | 1.40(0.88-2.10) | 0.353 |

ICU, intensive care unit; IQR, inter quartile range; APACHE Ⅱ score, acute physiology and chronic health evaluation Ⅱ score; SOFA, sequential organ failure assessment; *P* values were calculated by Mann-Whitney U test, Students’ t-test or one-way analysis of variance (one-way ANOVA), as appropriate. *P* values below 0.05 indicates statistical significance.
